# Feasibility and reliability of frailty assessment in the critically ill: a systematic review

**DOI:** 10.1186/s13054-018-1953-9

**Published:** 2018-02-26

**Authors:** Richard J. Pugh, Amy Ellison, Kate Pye, Christian P. Subbe, Chris M. Thorpe, Nazir I. Lone, Andrew Clegg

**Affiliations:** 1 Department of Anaesthetics, Glan Clwyd Hospital, Bodelwyddan, Denbighshire UK; 2 Bradford Teaching Hospitals NHS Foundation Trust, Bradford, UK; 3 School of Medical Sciences, Bangor University, Bangor, UK; 4 Department of Anaesthetics, Ysbyty Gwynedd, Bangor, UK; 5 Department of Anaesthesia, Critical Care and Pain Medicine, University of Edinburgh, Edinburgh, UK; 6 Academic Unit of Elderly Care and Rehabilitation, University of Leeds, Leeds, UK

**Keywords:** Frailty, Reliability, Feasibility, Clinical frailty scale, Frailty phenotype, Frailty Index, Critical care, Intensive care, Critically ill

## Abstract

**Background:**

For healthcare systems, an ageing population poses challenges in the delivery of equitable and effective care. Frailty assessment has the potential to improve care in the intensive care setting, but applying assessment tools in critical illness may be problematic. The aim of this systematic review was to evaluate evidence for the feasibility and reliability of frailty assessment in critical care.

**Methods:**

Our primary search was conducted in Medline, Medline In-process, EMBASE, CINAHL, PsycINFO, AMED, Cochrane Database of Systematic Reviews, and Web of Science (January 2001 to October 2017). We included observational studies reporting data on feasibility and reliability of frailty assessment in the critical care setting in patients 16 years and older. Feasibility was assessed in terms of timing of evaluation, the background, training and expertise required for assessors, and reliance upon proxy input. Reliability was assessed in terms of inter-rater reliability.

**Results:**

Data from 11 study publications are included, representing 8 study cohorts and 7761 patients. Proxy involvement in frailty assessment ranged from 58 to 100%. Feasibility data were not well-reported overall, but the exclusion rate due to lack of proxy availability ranged from 0 to 45%, the highest rate observed where family involvement was mandatory and the assessment tool relatively complex (frailty index, FI). Conventional elements of frailty phenotype (FP) assessment required modification prior to use in two studies. Clinical staff tended to use a simple judgement-based tool, the clinical frailty scale (CFS). Inter-rater reliability was reported in one study using the CFS and although a good level of agreement was observed between clinician assessments, this was a small and single-centre study.

**Conclusion:**

Though of unproven reliability in the critically ill, CFS was the tool used most widely by critical care clinical staff. Conventional FP assessment required modification for general application in critical care, and an FI-based assessment may be difficult to deliver by the critical care team on a routine basis. There is a high reliance on proxies for frailty assessment, and the reliability of frailty assessment tools in critical care needs further evaluation.

**Prospero registration number:**

CRD42016052073.

## Background

As the proportion of older patients admitted to critical care rises [1, 2], there is a pressing need to understand how critical care might best support a population with potentially complex medical, physical and psychosocial concerns. Only recently have studies started to explore the relevance of frailty assessment to the care of critically ill adults [3, 4]. Related to, though distinct from, co-morbidity and disability, frailty is a term used to describe "a condition characterised by loss of biological reserve and vulnerability to poor resolution of homeostasis following a stressor event" [5]. Frailty implies an impaired ability to withstand the physiological disturbance of an acute illness, and although it becomes more prevalent with age it is not exclusive to an older population.

A range of methods to evaluate frailty are described in the literature [5–8], with the utility of a particular frailty assessment tool dependent on the purpose, setting, time available, and skill of the assessor [9]. For the acutely ill, assessment tools might best be described as one of: a judgment-based measure (e.g. the clinical frailty scale (CFS) [10]); a single physical performance measure (e.g. grip strength); a frailty phenotype (depending on the presence of typically three to five criteria [11]); an extended multidimensional assessment (e.g. Tilburg Frailty Indicator [12, 13]); and a frailty index, the number of accumulated deficits associated with adverse outcome presented as a proportion with respect to the total number (30 or more) of pre-specified possible deficits [14].

Previous systematic reviews have examined the psychometric properties of frailty assessment tools in primarily non-acute settings [6–8, 15]. However, the validity and reliability of an assessment tool is largely dependent on the setting and population in which it was developed and validated [8], and frailty assessment in the critically ill poses particular challenges. At the outset of critical illness, there is often a reliance on proxy respondents [4]. Furthermore, frailty is itself an independent risk factor for delirium, can coexist with dementia and is associated with disability [5]. Proxy ratings do not necessarily correspond with a subject's own assessment of function or quality of life during the recovery from critical illness [16–19]. Last, there is inherent risk of inadvertently ascribing features of acute illness to underlying frailty [20] and of recall bias with retrospective inquiry. Given these concerns, we aimed to systematically review the literature to establish the feasibility and reliability of frailty assessment in the critically ill.

## Methods

Our review was performed according to recommendations for the systematic review of observational studies [21] and was registered prospectively via PROSPERO (PROSPERO, https://www.crd.york.ac.uk/PROSPERO/display_record.php?RecordID=52073) with registration number: CRD42016052073.

### Eligibility criteria

The inclusion criteria were:

1. The study included adult (16 years and over) patients being managed in a critical care environment.
2. The study involved the application of a multidimensional frailty assessment tool.
3. The study presented data relating to the feasibility of frailty assessment in the critically ill (timing of evaluation, the background, training and expertise required for assessors, and reliance upon proxy input), and/or of the reliability of frailty assessment in the critically ill.

Reviews, case reports and case series were excluded; studies that collected data retrospectively were not excluded, but the potential bias associated with retrospective as opposed to prospective study was considered. Data from the control arm of randomised controlled trials (RCTs) were considered for inclusion if the trial eligibility criteria identified a study population that was representative of the general critical care population. Studies were limited to English language publications from 2001 onwards. However, there was no restriction on the basis of publication status, provided eligibility criteria were otherwise met.

### Information sources

We searched the following databases from January 2001 to October 2017: Medline, Medline In-process, EMBASE, CINAHL, PsycINFO, AMED, Cochrane Database of Systematic Reviews, and Web of Science. Additional studies were sought from grey literature using the Open Grey database and by screening critical care conference abstracts, from the reference lists of papers and review articles, and through searches for full-text publication of relevant abstracts.

### Data management

Two authors independently screened titles and abstracts. An additional author also contributed to hand-searching reference lists of identified papers and review articles. A spreadsheet was used to keep a log of all potentially relevant studies and reasons for inclusion or exclusion. In the event of disagreement following full-text review, consensus was achieved through discussion without recourse to a third author. Data from included studies were extracted using a standardised data collection proforma and additional information sought from trial authors where appropriate.

### Data items

Study data extraction included: author, year, publication type, country, methodology and setting. Relevant patient characteristics of studied cohorts included: demographic data (age, gender), presence of co-morbidity, evidence of baseline level of dependence, primary reason for admission, surgical status, and severity of illness (according to established illness severity scales). Timing of frailty assessment was recorded (e.g. with reference to a point in time before the acute illness, at time of referral, at time of admission to critical care, or during recovery from acute illness), and interval between assessments. Information on the participation of the patient in the frailty assessment process was collected, as was the background of the individual(s) making the assessment of frailty.

### Outcomes and prioritisation

Feasibility of the frailty measurement tools in critical care was assessed on the basis of time taken to perform the assessment, training, and expertise required to implement, the proportion of potentially eligible patients excluded, and reasons for exclusion (e.g. due to lack of proxy).

Given that frailty state is not static in the context of an acute illness [4, 22], in assessing reliability our primary interest was the contemporaneous measure of inter-rater reliability. Where available, for ordinal scores we extracted the linear weighted kappa in line with consensus-based standards [23].

### Risk of bias for individual studies

We did not identify any eligible RCTs, but for observational studies the risk of bias was assessed using the Newcastle-Ottawa checklist [24] according to the domains of selection, comparability, exposure, and outcome. For each domain, a judgement of low, unclear, or high risk of bias was made. Studies were considered as at overall low risk of bias if all domains were judged as low risk; studies were considered high risk if any domains were deemed high risk. Depending on number and risk of studies identified, it was intended that sensitivity analysis might be performed excluding high-risk studies.

## Results

### Study selection

**Fig. 1:**
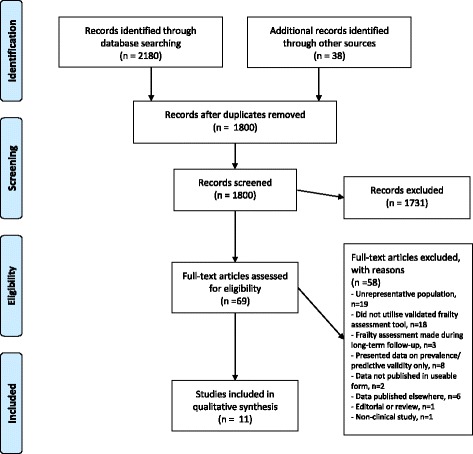
Flow diagram of included studies [25]

Search results are summarised in the Preferred Reporting Items for Systematic Reviews and Meta-Analyses (PRISMA) [25] diagram (Fig. 1). There were 2180 articles identified, of which 69 were considered potentially eligible. Following full-text review, a total of 11 study publications were included [26–36], representing 8 study cohorts and a total of 7761 patients.

### Study characteristics

The characteristics of included studies are summarised in Tables 1 and 2. Two research groups presented separate analyses of study cohorts in multiple publications (Bagshaw [26–28] and Heyland [33, 34]) but for the purposes of this review each study cohort was considered as a single study. Four studies were conducted in North America [26–29, 33–35], three in Europe (including one large study involving 21 countries) [30, 31, 36] and one in Australia [32]. The majority of studies were multi-centre [26–30, 33–36] and set in mixed medical-surgical ICUs. The median sample size was 385 patients (range 30–5021). Four of the studies adopted age restrictions: age 50 years or more [26–28], 65 years or more [30], and 80 years or more [33, 34, 36]. Only one study explicitly excluded patients with severe cognitive disease, including dementia [29]. The majority of patients were male, and 61% received invasive ventilation.

**Table 1:** Study characteristics

Study	Year	Full text	Country/countries	Number of sites	Setting	Intended study outcomes	Additional data provided by study authors?
Bagshaw [26–28]	2014, 2015, 2016	Yes	Canada	6	Mixed medical-surgical ICU	Predictive validity: in-hospital mortality, health-related quality of life	-
Brummel [29]	2017	Yes	USA	5	Medical and surgical ICU	Predictive validity: mortality, disability, cognitive impairment	Yes
Fisher [32]	2015	Yes	Australia	1	Mixed-medical surgical ICU	Predictive validity: mortality, length of stay, discharge destination Feasibility	-
Flaatten [36]	2017	Yes	Europe (21 countries)	311	Mixed ICUs	Predictive validity: mortality	Yes
Heyland [33, 34]	2015, 2015	Yes	Canada	22	Mixed ICUs	Predictive validity: prolonged dying experience, physical recovery at 12 months	Yes
Hope [35]	2017	Yes	USA	2	Medical and surgical ICUs	Construct validity: frailty markers, frailty assessment and demographic correlates of frailty. Predictive validity: new disability and death	Yes
Le Maguet [30]	2014	Yes	France	4	Mixed medical-surgical ICU	Predictive validity: mortality	Yes
Pugh [31]	2017	No - research letter only	UK	1	Mixed medical-surgical ICU	Inter-rater reliability of frailty assessment	Yes

**Table 2:** Patient characteristics

Study	Number of patients	Sex (% male)	Age restriction	Age in years, mean (+/- SD) or median (IQR)	Operative status	Proportion receiving invasive ventilation
Bagshaw [26–28]	421	61%	50 Years and older^a^	67 +/-10	Post-operative: 34%	86%
Brummel [29]	1040	60%	18 Years and older	62 (53- 72)	Post-operative: 16%	88%
Fisher [32]	348	59%^b^	18 Years and older	60 (+/-17)	Post-operative: 53%	Not reported
Flaatten [36]	5021	52%	80 Years and older	84 (81- 86)	Post-operative: 27%	51%
Heyland [33, 34]	610	55%	80 Years and older	84 (+/- 3)	Post-operative: 39%	72%
Hope [35]	95	56%	None	57 (+/- 18)	Post-operative: 6%	56%
Le Maguet [30]	196	65%	65 Years and older	75 (+/- 6)	Post-operative: 65%	88%
Pugh [31]	30	60%	16 Years and older	67 (+/- 14)	Not collected	Not collected

^a^Bagshaw (2016) focused on patients aged 50–64.9 years of age [28]

^b^Among patients undergoing clinical frailty scale assessment

In the majority of studies, the main aim was assessment of predictive validity (e.g. in terms of mortality, length of stay, disability, and health-related quality of life). In only one study was the primary intention to evaluate reliability of frailty assessment [31], and in only one other was the feasibility of frailty assessment an explicit outcome [32]. However, each of the other six studies included presented data sufficient to enable evaluation of the feasibility of frailty assessment in critical care.

### Risk of bias within studies

Six studies were considered at unclear overall risk of bias [26–31, 35, 36] and two studies were considered at low overall risk of bias [32–34] (Table 3). Regarding selection bias, the Bagshaw, Fisher, and Heyland reports were considered at low risk, since potential differences between patients recruited and not recruited had been explored [26–28, 32–34]. In terms of comparability, the Fisher, Heyland, and Hope reports were considered at low risk of bias, since in these studies the proxy contribution to assessment of frailty was quantified [32–35]. Only one study explored the reliability of frailty assessment, for which outcome bias was considered low risk due to the adoption of blinded assessment [31]. No studies were considered high risk and sensitivity analysis was therefore not performed.

**Table 3:** Risk of bias in included studies, with regards to feasibility and reliability

Study	Risk of bias				Notes
	Selection bias^a^	Comparability^b^	Outcome^c^	Overall risk	
Bagshaw [26–28]	Low	Unclear	n/a	Unclear	No statistically significant differences between study participants and non-enrolled. Involvement of proxy vs. patient not specified.
Brummel [29]	Unclear	Unclear	n/a	Unclear	Reasons for non-enrolment described, but potential differences between such patients not explored. Timing of frailty assessment described, but data regarding proxy involvement not collected.
Fisher [32]	Low	Low	n/a	Low	Reasons for not evaluating frailty not recorded, though there is comparison of evaluated vs. non-evaluated patients.
Flaatten [36]	Unclear	Unclear	n/a	Unclear	Numbers of potentially eligible patients not enrolled and reasons for non-enrolment not collated. Proxy data not collected.
Heyland [33, 34]	Low	Low	n/a	Low	Characteristics of study cohort were similar to unselected hospital cohort.
Hope [35]	Unclear	Low	n/a	Unclear	Reasons for non-enrolment described, but potential differences between such patients and those enrolled not studied. Proxy involvement described.
Le Maguet [30]	Unclear	Unclear	n/a	Unclear	Reasons for non-inclusion partially described. Potential differences between included and excluded patients not investigated.
Pugh [31]	Unclear	Unclear	Low	Unclear	Reasons for non-enrolment not recorded nor details regarding proxy involvement. Interval between assessments not recorded. Assessors were blinded to other assessments.

*n/a* not analysed

^a^Study group truly representative of critically ill population, reasons for non-inclusion are described, differences between included and excluded eligible patients analysed

^b^Description of proxy involvement, timing of assessments, interval between assessments, staff involved in assessments

^c^Investigating reliability of assessment, assessments performed independently and blindly analysed

### Assessment of frailty

**Fig. 2:**
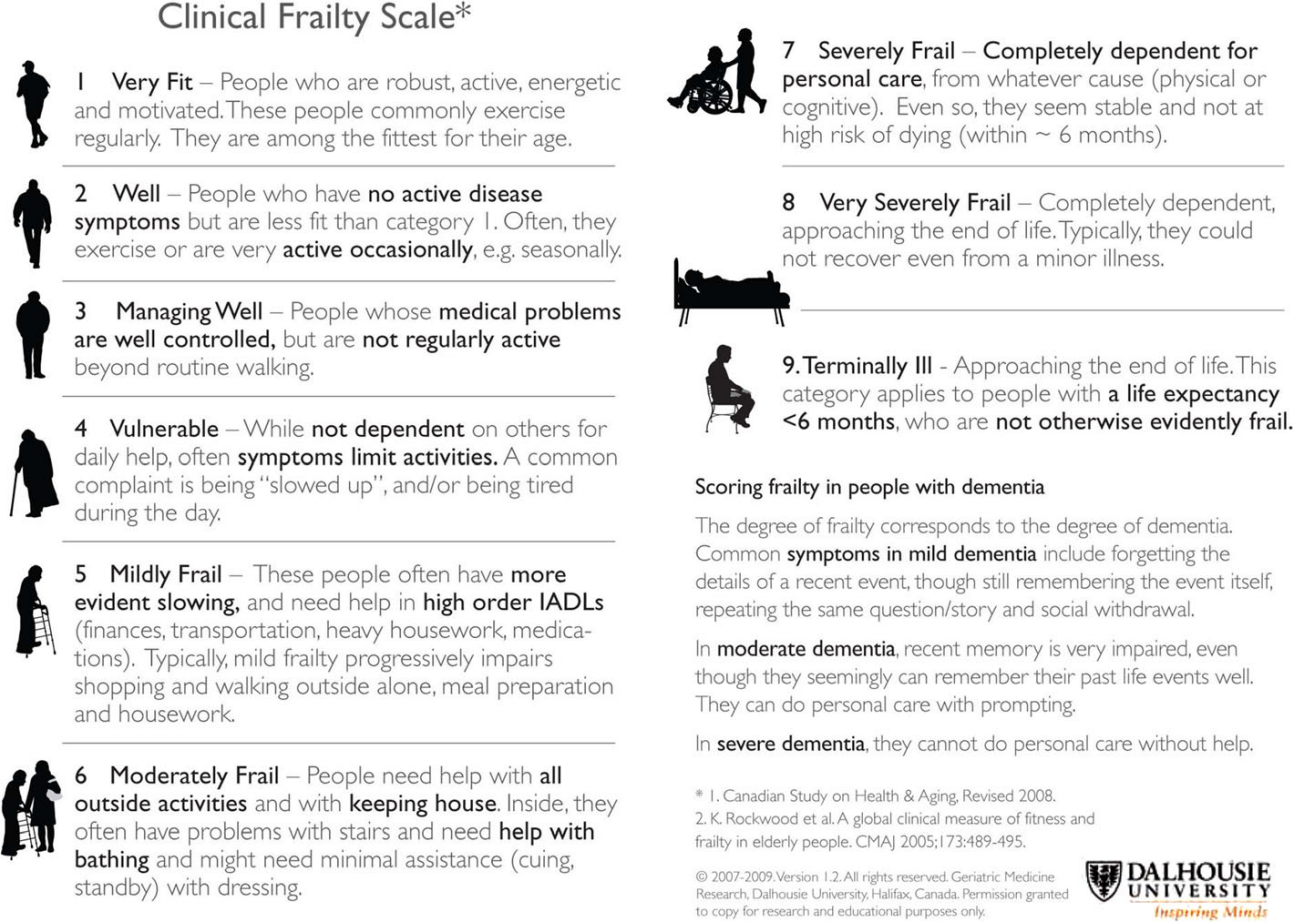
Clinical frailty scale [10]

All studies evaluated frailty using a clinical frailty scale (CFS) [10]; six studies used a 9-point scale [26–28, 30, 31, 35, 36] (see Fig. 2) and two a 7-point scale [29, 33, 34]. Additional scoring systems were used in three studies [30, 33–35]. Two studies utilised a frailty phenotype (FP) assessment, identifying frailty on the basis of the following domains: shrinking, weakness, slowness, low-level physical activity, and self-reported exhaustion [11], but modified for use in the critically ill (see Table 4) [30, 35]. Furthermore, Hope and colleagues supplemented these FP domains with questions relating to cognitive and to sensory impairment [35]. One study utilised a frailty index based on a 43-item comprehensive geriatric assessment (CGA) [33, 34].

**Table 4:** Assessment of frailty according to frailty phenotype

Frailty domain	Fried [11]	Le Maguet [30]	Hope [35]
Shrinking	Unintentional (not due to dieting or exercise) weight loss 10 lbs (4.5 kg) or more than 5% of body weight in the prior year	Unintentional (not due to dieting or exercise) weight loss ≥ 4.5 kg or more than 5% of body weight in the prior year	Reported weight loss and BMI < 24 or ≥ 5% weight loss
Weakness	Hand-grip strength measured by dynamometer (stratified by gender and body mass index)	Difficulty rising from a chair	Unable to rise from a chair without using arms
Slowness	Time to walk 15 feet (stratified by gender and height)	Slowed walking speed (during the last 6 months, with difficulties walking and with aid) and/or the occurrence of fall(s)	Falls or need for assistance with mobility inside or outside the home in the past year
Low physical activity	Use of Minnesota Leisure Time Activity Questionnaire to calculate kilocalories expended per week	Discontinued daily leisure activities such as walking or gardening and/or discontinued some sport activity per week	Unable to climb flight of stairs or undertake moderate activity, e.g. pushing a vacuum cleaner or bowling
Exhaustion	Feeling that everything the patient does is an effort and/or the feeling that he/she could not get going, and how often in the last 3 months he/she felt this way	Feeling that everything the patient does is an effort and/or the feeling that he/she could not get going, and how often in the last 3 months he/she felt this way	Feeling that everything the patient does is an effort and/or the feeling that he could not get going, in past 4 weeks; number of times he/she had a lot of energy in past 4 weeks
Cognitive Impairment			Memory Impairment Screen, or modified version of the Short-Form Informant Questionnaire on Cognitive Decline in the Elderly
Sensory Impairment			Problems in daily life because of poor vision or impaired hearing in last year

Frailty identified on the basis of two or three or more elements [35], or three or more elements [11, 30]

Timing of frailty assessment was available in seven studies. Frailty was assessed at critical care admission [30], within the first 24 hours [32, 36] or first 72 hours of critical care admission [29, 33–35] (Table 5). A variety of reference points for assessment were adopted: the subject's condition before ICU admission [32], before hospital admission [26–28], before onset of critical illness [29], before acute illness and hospital admission [36], 2 weeks before hospital admission [31, 33, 34] or one month before hospital admission [30].

**Table 5:** Issues relating to frailty assessment

Study	Frailty assessment tool	Timing of frailty assessment	Reference point for frailty assessment	Interval between assessments
Bagshaw [26–28]	CFS	Not recorded	Immediately before hospitalisation	Not applicable
Brummel [29]	CFS	Within 72 hours of ICU admission	Prior to critical illness	Not applicable
Fisher [32]	CFS	Within first 24 hours of ICU admission (for next-of-kin)	Pre-ICU admission	Not applicable
Flaatten [36]	CFS	Within first 24 hours of ICU admission	Before acute illness and hospital admission	Not applicable
Heyland [33, 34]	CFS, FI	At 48 − 72 hours after ICU admission	At 2 weeks pre-hospital admission	Not recorded
Hope [35]	CFS, FP	Within 72 hours of ICU admission	CFS: not specified FP: variable, depending on element	Within 24 hours
Le Maguet [30]	CFS, FP	At ICU admission	At 1 month pre-hospital admission	Not recorded
Pugh [31]	CFS	Not recorded	At 2 weeks pre- hospital admission	Not recorded

*FP* frailty phenotype, *CFS* clinical frailty score, *n/a* not analysed

### Feasibility of frailty assessment

A member of the research team assessed frailty in five studies [26–29, 31, 33–35]; in three studies critical care doctors with clinical rather than research responsibilities assessed frailty using a CFS [31, 35] or a CFS and FP [30]; in one study critical care nurses and doctors assessed frailty using a CFS [36], and in one study it was the nurse-in-charge or next-of-kin (with nurse guidance) who assigned a CFS score [32] (Table 6). Researchers received specific training to familiarise themselves with the study and assessment tool(s) in four studies [29, 31, 33–35]; critical care nurses received CFS training in the form of a series of lectures in one study [32]. In the remaining three studies, training clinical staff to use CFS took place in a staff meeting [30], at the bedside [31, 35] or was deemed not to need particular training "since the description combined with illustration is intuitive" [36]. The times required for training and to perform assessments were for the most part not captured during the course of included studies (Table 6).

**Table 6:** Feasibility of frailty assessment

Study	Frailty assessor(s)	Training required	Time required for assessment	Patient participation	Proxy involvement	Neither patient nor proxy involved	Percent screened patients^a^ excluded due to lack of proxy
Bagshaw [26–28]	Research coordinator	Not described	Not recorded	Not recorded	Not recorded	Nil	Not recorded
Brummel [29]	Study personnel	Trained by geriatrician during 2-day trial start up	Not recorded	Not recorded	Not recorded	Nil	31% because of lack of proxy
Fisher [32]	Next-of-kin (NOK), nurse in charge if NOK unavailable in first 24 hours	Series of lectures for nurses; bedside introduction with standardised script for NOK	Not recorded	None	73%	27%	Nil
Flaatten [36]	Critical care staff	None	Not recorded	Not recorded	Not recorded	Not recorded	< 0.2% because of lack of proxy^b^
Heyland [33, 34]	Research coordinator	Embedded within start-up training	Not recorded	None	100% (excluded if no family member available)	Nil	45% missed caregiver
Hope [35]	CFS: critical care doctor FP: research team	Bedside explanation (CFS) Specific training in use of questionnaire and frailty construct (FP)	Not recorded	42%	58%	Nil	12% because of lack of proxy
Le Maguet [30]	Critical care staff	Staff meeting	Not recorded	39%	69%	Nil	20% because of lack of proxy
Pugh [31]	Medical student investigators, critical care doctor	Training for medical students, bedside explanation for doctors.	Not recorded	Not recorded	Not recorded	Nil	Not recorded

*CFS* clinical frailty scale, *FP* frailty phenotype

^a^Otherwise meeting study enrolment criteria

^b^Not specified by authors but 99.8% included patients have frailty assessment

Two studies excluded patient involvement in frailty assessment in their methodology [32–34]. Of these, availability of a family member was a requirement for study enrolment in Heyland's study [33, 34]. In Fisher's study, the CFS assessment was expected to be made by the next-of-kin under the guidance of the bedside nurse, using a standardised introduction; however, CFS assessment was made by the nurse-in-charge if the next-of-kin was unavailable in the first 24 hours of critical care admission [32]. The researchers reported that when no assessment had been made, patients were more likely to have been post-operative or to have had a shorter critical care stay. In Le Maguet [30], 31% of patients were able to interact with an interviewer to enable assessment, and in Hope [35], 42% patients contributed to frailty assessment. In the other three studies, patient participation was not recorded.

Where recorded, a proxy was involved in 58% [35], 69% [30], 73% [32] and 100% [33, 34] of frailty assessments (Table 6). Fisher found that it was not possible to approach the next of kin for involvement in assessment in 27% cases within the first 24 hours of critical care admission; however, since the nurse in charge could make a CFS assessment on the basis of medical records, this did not prohibit frailty assessment [32]. Flaatten did not specify the contribution of patient or proxy to assessment, but noted that CFS assessment was achieved in 99.8% of cases included [36]. Hope noted that in some instances surrogates were unable to answer questions relating to an assessment of frailty according to FP assessment, for example, according to domains of weight loss (5% of cases) or loss of energy (3% of cases) [35]. Le Maguet also found that "several components of the FP score, notably those that evaluate performance, were difficult to explore in ICU patients" [30]. Overall, screened patients excluded from enrolment due to lack of proxy availability ranged from 0 to 45% in five studies [29, 30, 32–35]: the highest exclusion rate was in Heyland's study, in which frailty was assessed according to a 43-item CGA and a CFS, and in which family involvement was an absolute requirement for enrolment [33, 34].

### Reliability of frailty assessment

Reliability of frailty assessment was assessed in only one study, which evaluated the inter-rater reliability of frailty assessment using a CFS as a comparison between two groups, a group of medical students and a group of critical care doctors. Linear weighted kappa was 0.64 (95% confidence intervals 0.40 to 0.87, *p* < 0.0001), suggesting good agreement. However, this was a small (*n* = 30), single-centre study comparing only two groups of assessors (medical students and critical care doctors, excluding other members of the clinical team), and which did not make reference to the relative contributions of the critical care patient or a proxy.

## Discussion

In assessing the feasibility of frailty assessment, we have made a distinction between those primarily involved in clinical and in research roles in view of anticipated differences in training and time available to apply assessment tools. Though not well-described, clinical staff for the most part appear to have received relatively little training with regards the application of frailty assessment tools compared with those described as "research coordinator" or "study personnel." Despite this, the high proportion of patients among included studies undergoing frailty assessment using the judgement-based CFS by clinical staff is likely to reflect its simplicity and ease of application [36]. Indeed CFS assessment seemed achievable even in the absence of family contact [32].

With regard to other assessment methods, the two studies utilising FP assessment reported difficulties with some components of FP assessment, despite making adaptations for a critically ill population. However, FP assessment was used by both clinical [30] and research staff [35]. The early phase of critical illness typically precludes elements of frailty assessment, which require demonstration rather than description (e.g. grip strength and gait speed), though such assessment appears feasible for ICU survivors much nearer to hospital discharge [37]. The consequences of such modifications for frailty classification and predictive validity in this population are uncertain [38]. FI-CGA was used to assess frailty in only one study, and although the time required to administer the 43-item questionnaire to a family member by the research coordinator was not recorded, it is unclear whether this would be too time-consuming to be feasibly delivered by a critical care team on a routine basis. Recent research has reported the development and validation of a 36-item electronic frailty index (eFI) using routinely available electronic medical record (EMR) data [39], which may be an attractive approach for critical care but requires further validation in this context.

A high proportion of enrolled patients were invasively ventilated and only a minority able to participate directly in frailty assessment. There is clearly a heavy reliance on proxy input for frailty assessment in this population, particularly when detailed information is required [32–34], and Heyland's study illustrates the difficulty of coordinating the availability of assessor and an appropriate family member when FI-CGA assessment is made by a limited number of trained individuals.

A dependence on proxy input is also highly relevant when considering the reliability of frailty assessment in the critically ill. We identified only one small clinical study that investigated the inter-rater reliability of CFS assessment between a group of critical care doctors and a group of medical students [31]. Although there was a good level of agreement, the study did not capture the relative contributions of the patient or their proxies to frailty assessment. Furthermore, we found no study that had attempted to compare assessment of frailty between clinical staff and critically ill patients or their relatives, or to evaluate the influence of clinical background, seniority, and training on frailty assessment. When carefully selected, other investigators have identified a high level of agreement between subject and proxy in terms of functional status after critical illness [40]. However, the retrospective nature of frailty assessment (in the manner identified in included studies) makes it prone to recall bias, particularly in the context of acute and sub-acute chronic illness [14, 41]. Furthermore, subjective elements (e.g. "exhaustion" [30, 35]) rather than observable criteria may be especially susceptible to differences between proxy and subject ratings [42, 43]. The reliability of frailty assessment by clinicians is an important issue; in other settings, escalation decision-making following emergency admission may be made on the basis of a perception of baseline cognitive and functional status, which at best only modestly correlates with that of patient or relative [44].

There are several strengths to this review. We prospectively registered our review protocol and have followed rigorous methodology to identify, evaluate, and summarise the current evidence on feasibility and reliability of frailty assessments in the critically ill. However, we recognise some limitations. Assessment of the feasibility and reliability of frailty assessment was the intended outcome of only two of the eight studies included, and this is reflected in absent or incomplete data on factors that may have contributed to inclusion or exclusion of patients (e.g. to "missed caregiver"), the background of assessor, the training and time taken to perform assessment, background of any proxies, and the relative involvement of patient and proxy in the assessments. As a consequence, for the purposes of evaluating feasibility and/or reliability of frailty assessment in the critically ill, only two studies were considered at low overall risk of bias.

However, our review has a number of implications for clinical practice. We found evidence that frailty assessment can feasibly be performed by different clinical members of the critical care team (whether physician, nurse or medical student), that patient participation in such assessment will be achieved in a minority of cases, and that a qualifying proxy is usually required. We found limited data indicating that frailty can be reliably assessed by clinicians in the critical care setting. Given the challenges inherent in frailty assessment in critical illness, more research is needed regarding the reliability of frailty assessment tools in critical care before frailty assessment can be used to aid clinical decision-making and/or trigger interventions.

This review highlights areas for future research. Frailty is a dynamic state, and frailty assessment in the context of variations in health trajectories prior to critical illness needs exploration. Further study is required to compare the relative performance of frailty assessment tools in critical care, taking into account the reference point for assessment, the background and training of the assessor(s), the capacity of the patient, and the relationship between patient and proxy. In particular, the relative performance of frailty assessment using routinely captured data versus bedside frailty assessment should be evaluated in this population. Last, a clearer understanding of the training required and the time taken to make an assessment of frailty needs to be considered in the context of the potential benefits of making that assessment.

## Conclusions

This review has found little evidence of reliability and only limited evidence on the feasibility of frailty assessment in the critically ill. CFS was the most widely applied assessment tool by clinical staff, conventional FP assessment required modification for general application in critical care, and FI-based assessment may be difficult to deliver by the critical care team on a routine basis. Additional research is required to investigate the resource implications of routine use of frailty tools, to evaluate reliability when used by a range of clinical personnel, to investigate the use of routinely available EMR data for identifying frailty, and to study reliability in the presence or absence of clinical proxies before recommending widespread application in routine critical care practice.

## Abbreviations

ADLs: Activities of daily living
CFS: Clinical frailty scale
CGA: Comprehensive geriatric assessment
eFI: Electronic frailty index
EMR: Electronic medical record
FI: Frailty index
FP: Frailty phenotype
ICU: Intensive care unit
IQR: Interquartile range
PRISMA: Preferred Reporting Items for Systematic Reviews and Meta-Analyses
SD: Standard deviation
TFI: Tilburg Frailty Indicator
